# Supplementary figures and images for: EBV Triggers a Distinct Antiviral Response in HMC3 Cells
Source: bioRxiv. 2026 Apr 7:2026.04.03.716358. Preprint. [Version 1] doi: 10.64898/2026.04.03.716358 (PMC13082142; doi:10.64898/2026.04.03.716358)

## Slide 1
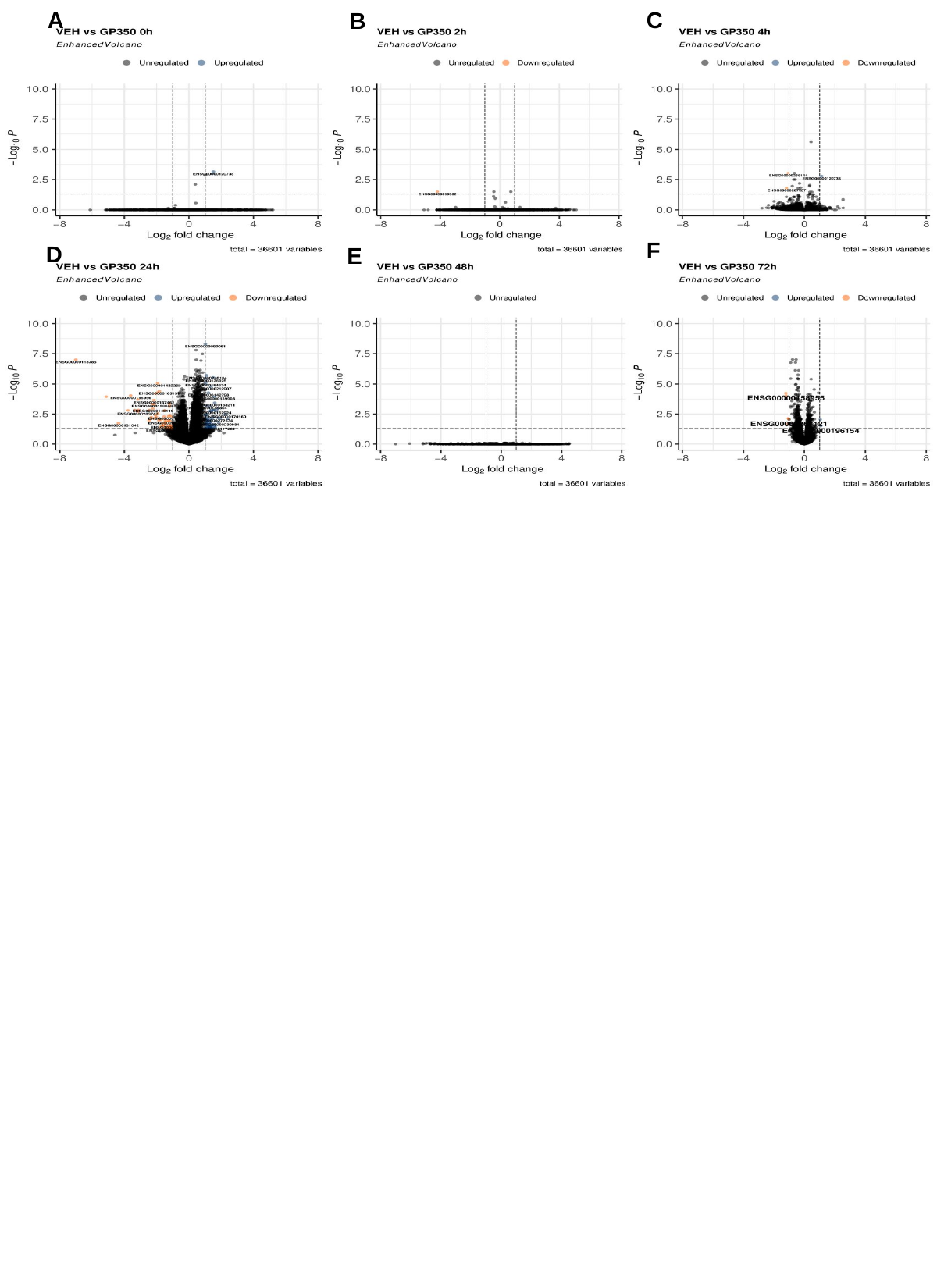

A
C
B
F
D
E

## Slide 2
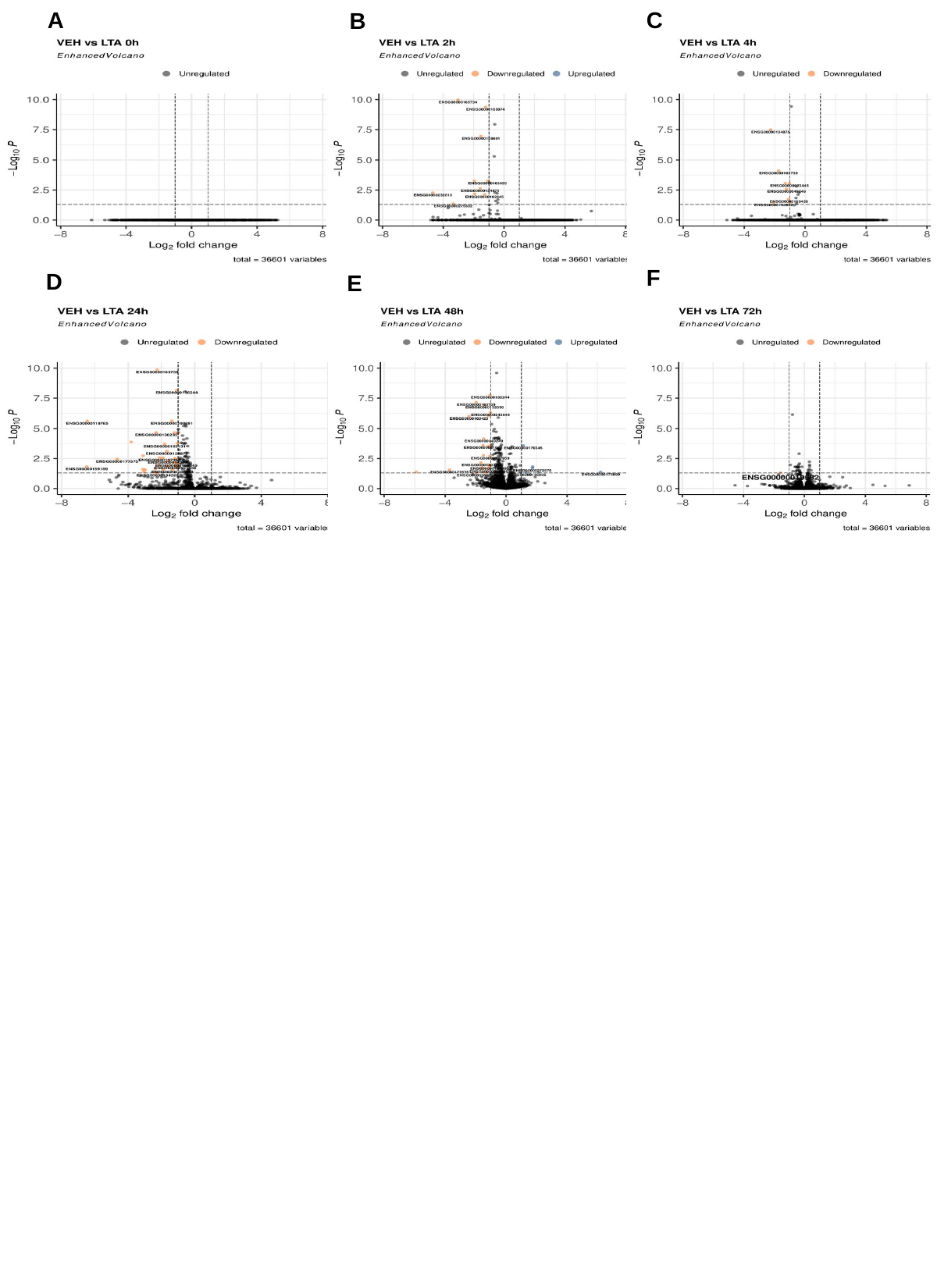

A
C
B
F
D
E

## Slide 3
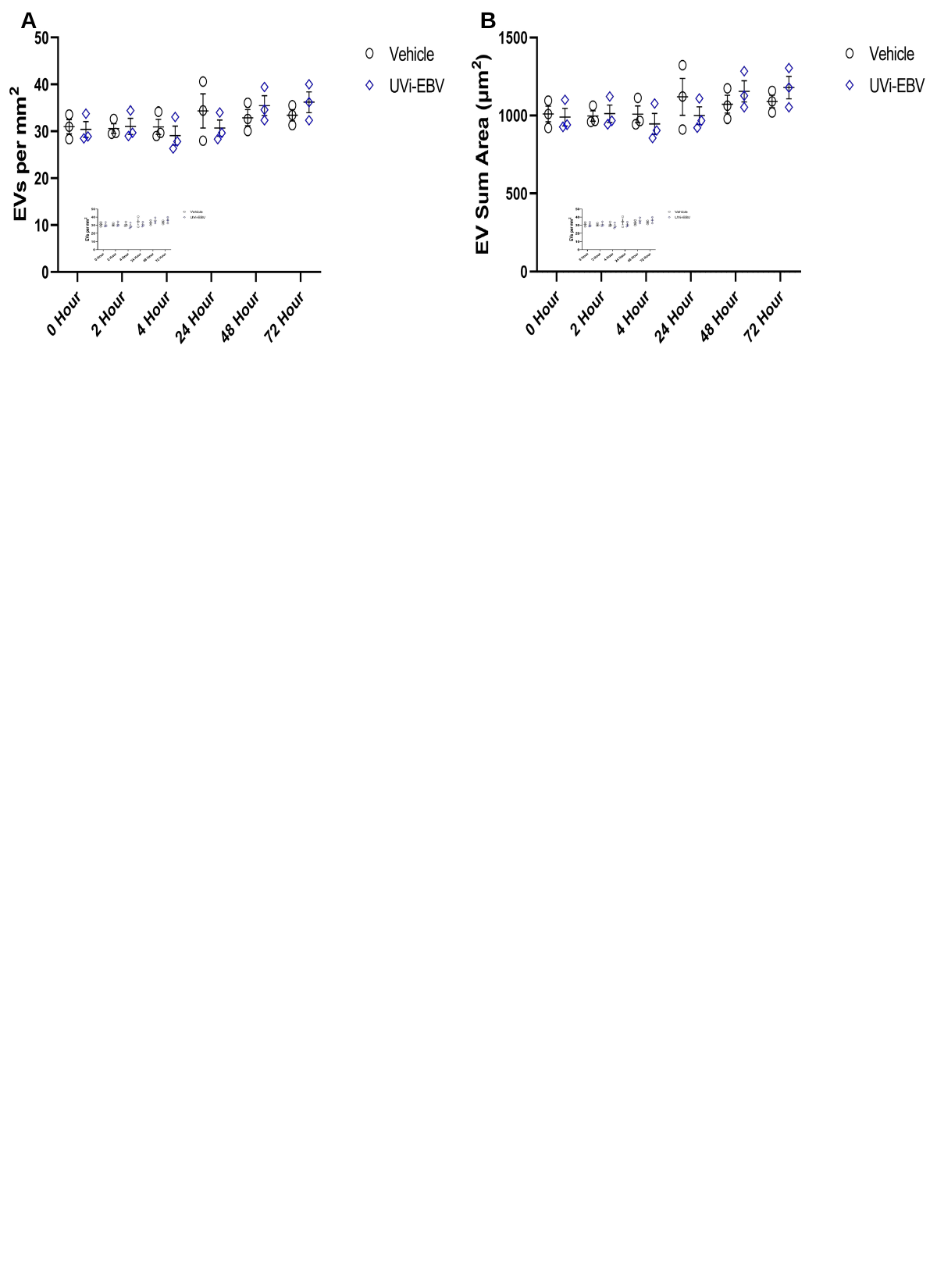

A
B

Supplement: Supplement 1 — Supplemental Figure 1: GP350 Regulation of Microglial Gene Expression Outputs of RNA sequencing from the transcriptomes of HMC3 cells treated with VEH or GP350 (VEH+80ng/mL). Volcano plots exhibit differential expression patterns of human genome build 38 (hg38) annotated loci between VEH and UVi-EBV treated samples at (A) 0-, (B) 2-, (C) 4-, (D) 24-, (E) 48-, (F) and 72-hours post-exposure. Differential expression was determined with a z score using shrunken estimates of log fold change divided by standard error and a Benjamini-Hochberg correction of p values (padj). Loci were determined as differentially expressed when possessing an absolute fold change >1 and a padj <0.05. Orange dots indicate loci downregulated in GP350 exposure, blue dots indicate loci upregulated in GP350 exposure, and grey dots indicate unchanged loci. Supplemental Figure 2: LTA Regulation of Microglial Gene Expression Outputs of RNA sequencing from the transcriptomes of HMC3 cells treated with VEH or LTA (VEH+1μg/mL). Volcano plots exhibit differential expression patterns of human genome build 38 (hg38) annotated loci between VEH and UVi-EBV treated samples at (A) 0-, (B) 2-, (C) 4-, (D) 24-, (E) 48-, (F) and 72-hours post-exposure. Differential expression was determined with a z score using shrunken estimates of log fold change divided by standard error and a Benjamini-Hochberg correction of p values (padj). Loci were determined as differentially expressed when possessing an absolute fold change >1 and a padj <0.05. Orange dots indicate loci downregulated in LTA exposure, blue dots indicate loci upregulated in LTA exposure, and grey dots indicate unchanged loci. Supplemental Figure 3: UVi-EBV Does Not Influence Microglial Endocytosis Measures of pHrodo™ Green conjugated 10kDa Dextran beads via the FitC channel at 0-, 2-, 4-, 24-, 48-, and imaged via a 6,441μm in diameter circle (32.58mm2 area). The extent of microglial endocytosis was recorded in the number of endocytic vesicles (EVs) per 1m [file media-1.pptx]
